# Expression analysis of G Protein-Coupled Receptors in mouse macrophages

**DOI:** 10.1186/1745-7580-4-5

**Published:** 2008-04-29

**Authors:** Jane E Lattin, Kate Schroder, Andrew I Su, John R Walker, Jie Zhang, Tim Wiltshire, Kaoru Saijo, Christopher K Glass, David A Hume, Stuart Kellie, Matthew J Sweet

**Affiliations:** 1 Cooperative Research Centre for Chronic Inflammatory Diseases and Special Research Centre for Functional and Applied Genomics, Institute for Molecular Bioscience, Institute for Molecular Bioscience, University of Queensland, Brisbane, Queensland, 4072, Australia; 2 The Genomics Institute of the Novartis Research Foundation, San Diego, CA 92121, USA; 3 The Department of Cellular and Molecular Medicine, University of California, San Diego, La Jolla, CA 92093-0651, USA; 4 The Roslin Institute, University of Edinburgh, Roslin EH25 9PS, Scotland, UK; 5 School of Molecular and Microbial Sciences, University of Queensland, Brisbane, Queensland, 4072, Australia

## Abstract

**Background:**

Monocytes and macrophages express an extensive repertoire of G Protein-Coupled Receptors (GPCRs) that regulate inflammation and immunity. In this study we performed a systematic micro-array analysis of GPCR expression in primary mouse macrophages to identify family members that are either enriched in macrophages compared to a panel of other cell types, or are regulated by an inflammatory stimulus, the bacterial product lipopolysaccharide (LPS).

**Results:**

Several members of the P2RY family had striking expression patterns in macrophages; *P2ry6 *mRNA was essentially expressed in a macrophage-specific fashion, whilst *P2ry1 *and *P2ry5 *mRNA levels were strongly down-regulated by LPS. Expression of several other GPCRs was either restricted to macrophages (e.g. *Gpr84*) or to both macrophages and neural tissues (e.g. *P2ry12*, *Gpr85*). The GPCR repertoire expressed by bone marrow-derived macrophages and thioglycollate-elicited peritoneal macrophages had some commonality, but there were also several GPCRs preferentially expressed by either cell population.

**Conclusion:**

The constitutive or regulated expression in macrophages of several GPCRs identified in this study has not previously been described. Future studies on such GPCRs and their agonists are likely to provide important insights into macrophage biology, as well as novel inflammatory pathways that could be future targets for drug discovery.

## Background

Macrophages are key cellular mediators of acute and chronic inflammation. They can be defined on the basis of morphology, function (e.g. non-specific uptake of particles) and expression of specific cell surface markers (e.g. EMR1 that is detected by the F4/80 antibody). EMR1 belongs to the GPCR super-family [1], and it is clear that macrophages express a diverse repertoire of other GPCRs [2]. A large number of GPCRs are yet to be deorphanized and hence, there is great potential for this family in both the discovery of gene function and in drug development. In this study we performed a systematic micro-array analysis of GPCR expression in primary mouse macrophages and identified family members that are either enriched in macrophages compared to a panel of other cell types, or are regulated by the pro-inflammatory stimulus, bacterial LPS. Some of these GPCRs are likely to provide future targets for drug discovery in inflammatory disease settings.

## Results

### Constitutive GPCR expression in mouse macrophages

Micro-array expression profiling across a panel of mouse and human tissues has enabled detailed analysis of tissue-specific gene expression [3]. In this study, we analyzed the constitutive and regulated expression of GPCRs in macrophages by interrogating a more detailed micro-array dataset of 91 murine cell types and tissues [3,4]. This data, available through NCBIs Gene Expression Omnibus ([5] accession number GSE10246), can also be used to probe gene expression profiles for an extensive array of other cell types.

The cell and tissue panel included two of the most commonly used primary mouse macrophage models; bone marrow-derived macrophages (BMM) and thioglycollate-elicited peritoneal macrophages (TEPM), as well as microglia (a resident tissue macrophage population of the brain) and the widely utilized macrophage-like cell line, RAW264. We analyzed the expression patterns of all GPCRs as defined by the International Union of Basic and Clinical Pharmacology [6,7]. We then compared the GPCR expression profiles of BMM and TEPM to the remaining panel of cell types, and in doing so, identified 67 GPCRs that were detectably expressed either constitutively or in an LPS-induced manner in these primary macrophage populations [see additional file 1].

**Figure 1:**
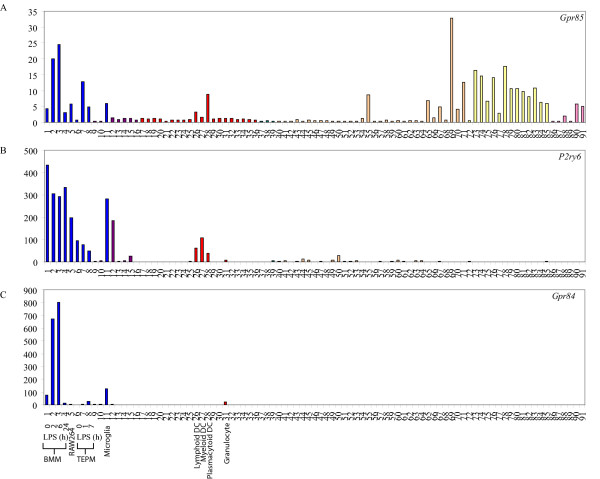
**Macrophage-restricted GPCRs**. Micro-array analysis of *Gpr85 *(A), *P2ry6 *(B) and *Gpr84 *(C) mRNA expression across a panel of 91 murine cell types and tissues. Data points show normalised values and similar cell types are grouped according to bar colour; blue indicates primary macrophage cell types, purple indicates bone-related cell types, red indicates other immune cell types, green indicates stem cell populations, orange indicates whole tissue samples, yellow indicates neuronal and retinal cell types and pink indicates cell lines. Additional file 3 gives details of the 91 cell types and tissues profiled.

In order to identify GPCRs that were very highly expressed by BMM and/or TEPM, we set an arbitrary cut-off in which normalised expression in either BMM or TEPM was at least 10-fold greater than the median normalised expression across all cell types. Using this very strict filtering approach, we identified 33 GPCRs that were enriched in BMM and/or TEPM as compared to the median normalised expression (Table 1 and 2). This, of course, does not mean that such genes are exclusively expressed by macrophages. For example, *Gpr85 *was highly expressed in several macrophage populations and was further up-regulated by LPS, but was also expressed in several regions of the brain (Figure 1a). Nevertheless, included in this set were GPCRs such as *Emr1 *(*F4/80*), *C3aR*, *C5aR*, *Ccrl2 *and *Ccr2 *that are known to be either macrophage-specific or highly expressed by macrophages [8-12], thus validating our approach. Also included in this list were a number of GPCRs (e.g. *Edg5*, *P2ry2 *and *6*) that have not previously been reported to have a macrophage-restricted expression pattern (Table 2). As demonstrated in figure 1b, *P2ry6 *expression was strikingly restricted to macrophages and two cell types closely related to macrophages (osteoclasts and dendritic cells), whilst a number of other P2Y family receptors were also highly expressed by this lineage (Table 2). Consistent with this conclusion, we recently observed a macrophage-restricted expression pattern for *P2ry6 *mRNA in a much more limited analysis across seven different cell populations [2]. *Gpr84 *expression was also largely restricted to macrophage populations and granulocytes (Figure 1c).

**Table 1:** Summary of GPCRs expressed by macrophages.

**Highly Expressed**	
**BMM**	**TEPM**

22 10 only enriched in BMM	23 11 only enriched in TEPM
12 regulated in both BMM and TEPM	

**LPS Regulation**	

**Induced**	

BMM	TEPM
27 14 only regulated in BMM	16 3 only regulated in TEPM
13 regulated in both BMM and TEPM	

**Repressed**	

BMM	TEPM
19 9 regulated only in BMM	18 8 regulated only in TEPM
10 regulated in both BMM and TEPM	

Summary of GPCRs highly expressed or regulated in macrophages as assessed by micro-array analysis.

**Table 2:** Macrophage-enriched GPCRs.

			**Relative Normalised Expression**		
			
**Receptor Class**	**Accession Number**	**Gene Name**	**BMM**	**TEPM**	**RAW264.7**
**Enriched in both BMM and TEPM**					

**Class A**					
	NM_009779	C3ar1	1361.1	683.6	58.3
	NM_007577	C5r1	822.6	25.6	10.5
	NM_183168	P2ry6	434.0	94.0	196.4
	NM_009917	Ccr5	214.2	20.5	0.6
	NM_021476	Cysltr1	208.9	12.5	40.6
	NM_004230	Edg5	30.2	69.3	6.6
	NM_008967	Ptgir	24.7	72.1	1.7
	NM_009924	Cnr2	14.2	27.2	6.7
	NM_009912	Ccr1	14.1	235.1	16.3
	NM_145700	Ccrl1	11.1	26.6	12.7
	NM_008964	Ptger2	10.7	34.4	0.8
**Class B**					
	NM_010130	Emr1	190	22.2	28.3

**Enriched in BMM**					

**Class A**					
	NM_183031	Ebi2	113.5	6.1	245.2
	NM_009915	Ccr2	92.1	1.2	1.3
	NM_030720	Gpr84	77.5	0.6	2.7
	NM_008042	Fprl1	32.0	1.0	0.8
	NM_008152	Gpr65	29.3	9	6.6
	NM_009910	Cxcr3	26.3	1.1	7.6
	NM_007420	Adrb2	22.1	1.3	9.3
	NM_145066	Gpr85	13.7	1.7	7.6
	NM_009987	Cx3cr1	11.3	0.7	61.4
**Class B**					
	NM_018782	Calcrl	45.6	1.1	6.1

**Enriched in TEPM**					

**Class A**	NM_009911	Cxcr4	4.3	178.9	0.6
	NM_017466	Ccrl2	3.1	154.9	1.1
	NM_021381	Prokr1	1.0	36.6	0.7
	NM_022320	Gpr35	2.5	22.0	0.4
	NM_008311	Htr2b	1.1	19.9	0.5
	NM_008773	P2ry2	8.5	19.2	1.5
	NM_133200	P2ry14	4.0	18.4	0.4
	NM_175493	Gpr68	0.9	17.9	9.3
	NM_008772	P2ry1	4.4	10.6	0.6
**Class B**					
	NM_011925	Cd97	0.4	22.9	0.2
**Class C**					
	NM_022420	Gprc5b	0.6	60.6	0.6

Micro-array analysis was used to identify GPCRs that were highly enriched in primary mouse macrophages (BMM and TEPM). GPCRs identified as highly enriched in BMM or TEPM were defined as GPCRs where the normalised value was as least 10-fold greater and significantly different (p-value < 0.05, Student's two-tailed t-test) from the median expression across all cell types. GPCRs are grouped according to their class type on the basis of sequence similarity as previously described [65-67].

### Differential GPCR expression in distinct macrophage populations

**Figure 2:**
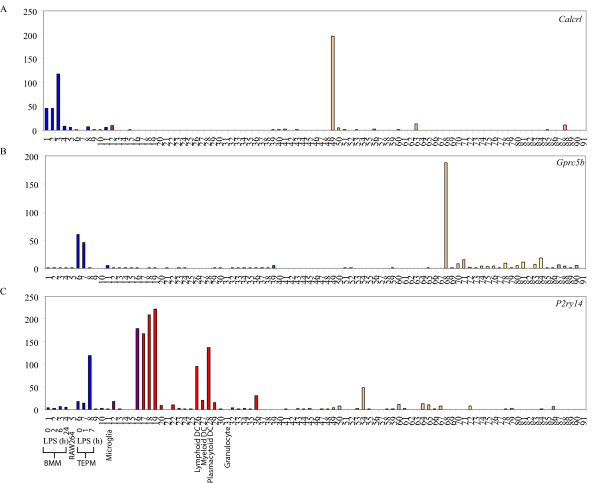
**GPCRs differentially regulated in BMM and TEPM**. Micro-array analysis of *Calcrl *(A), *Gprc5b *(B) and *P2ry14 *(C) mRNA expression across a panel of 91 murine cell types and tissues. Data points show normalised values and similar cell types are grouped according to bar colour; blue indicates primary macrophage cell types, purple indicates bone-related cell types, red indicates other immune cell types, green indicates stem cell populations, orange indicates whole tissue samples, yellow indicates neuronal and retinal cell types and pink indicates cell lines. Additional file 3 gives details of the 91 cell types and tissues profiled.

We also searched for GPCRs with differing expression patterns in non-inflammatory macrophages (BMM) versus inflammatory macrophages (TEPM). Those GPCRs that were highly expressed by BMM, but not TEPM, included *Ebi2*, *Calcrl*, *Ccr2 *and *5*, *Cxcr3*, *Cx3cr1 *and *Gpr84*, whilst TEPM expressed much higher levels of *Cxcr4*, *Gpr35*, *P2ry1*, *Ccr1*, *Cd97*, *Ccrl2 *and *Gprc5b *(Table 2). As an example, figure 2a and 2b demonstrate the selective expression of *Calcrl *and *Gprc5b *in BMM and TEPM, respectively. The divergent expression patterns could, of course, reflect the differential growth state of the cells; BMM are cycling cells whilst TEPM are post-proliferative. Indeed, many of these genes showed similar expression profiles in BMM and RAW264 cells, both of which are proliferating macrophage populations. Nonetheless, there were also GPCRs that had distinct expression levels in BMM versus TEPM, as well as BMM versus RAW264. It is therefore unlikely that the differential expression of these genes in BMM versus TEPM is a reflection of the proliferative state of the cells. Examples of these include *Ccr2*, *Gpr84 *and *Fprl1 *that were selectively enriched in BMM as compared to TEPM and RAW264, whilst *Gpr68 *was enriched in both TEPM and RAW264 but not BMM. Such GPCRs may have distinct functions in macrophage differentiation, recruitment or activation. Apart from our analysis of macrophage-enriched GPCRs, we also identified several GPCRs (e.g. *Gpr65*, *Ccr1*, *Ccrl1*, *Cnr2 *and *P2ry14*) that were highly expressed by multiple leukocyte populations. As an illustration, figure 2c shows the immune-restricted expression of *P2ry14*.

### LPS-regulated GPCR expression in mouse macrophages

**Figure 3:**
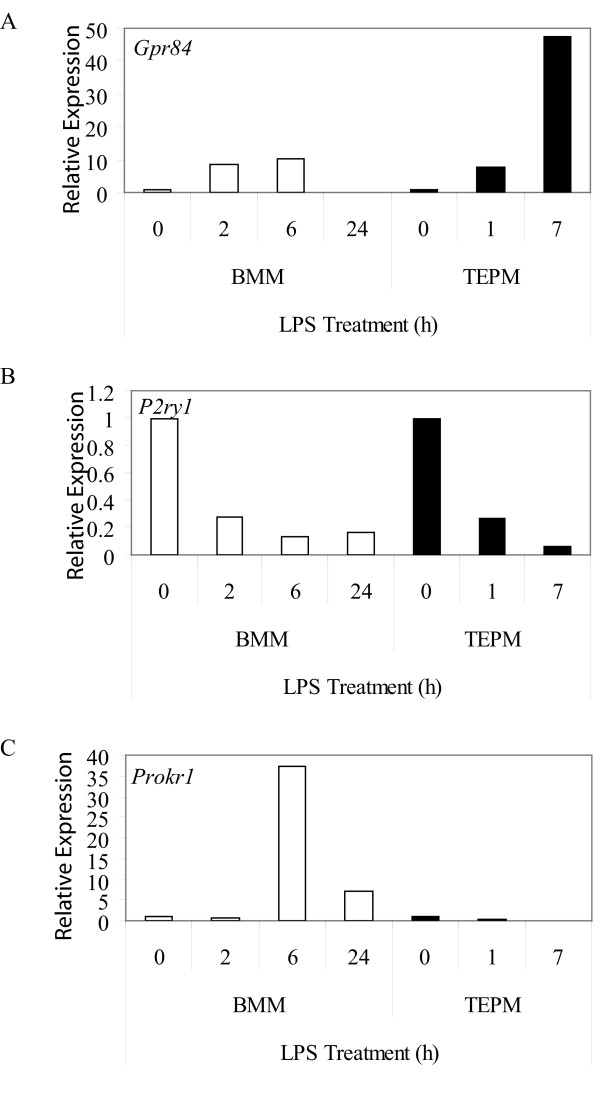
**GPCRs regulated by LPS in macrophages**. Micro-array expression analysis of *Gpr84 *(A), *P2ry1 *(B) and *Prokr1 *(C) mRNA in BMM and TEPM over a timecourse of 0, 2, 6, and 24 h, and 0, 1 and 7 h, respectively. Data points show gene expression relative to untreated control for each cell population (0 h).

LPS, the archetypal macrophage-activating stimulus that signals via toll-like receptor (TLR) 4, regulated the expression of several GPCRs in macrophages (Table 3 and 4). Whilst LPS is already known to regulate the expression of many of these (e.g. *Gpr109b*/*PUMA-G*, *Fpr1*, *Ccrl2 *and *Ednrb*) ([12-16], Sweet *et al*, unpublished data), regulated expression of others has not been widely studied. Examples of these include *Fzd1*, *Htr2b*, *Gpr84 *(induced by LPS, Table 3), and *P2ry1 *and *5 *(repressed by LPS, Table 4). We recently validated the LPS-mediated repression of *P2ry5 *mRNA expression in BMM by real time PCR [2]. Figure 3 highlights two examples of regulated GPCR expression: mRNA expression of *Gpr84 *was induced 10-fold by LPS (6 h) in BMM and 48-fold by LPS (7 h) in TEPM (Figure 3a), whilst *P2ry1 *was repressed 7-fold by LPS (6 h) in BMM and 18-fold by LPS (7 h) in TEPM (Figure 3b).

**Table 3:** GPCRs induced by LPS in macrophages.

			**Relative Normalised Expression**				
			
**Receptor Class**	**Accession Number**	**Gene Name**	**BMM**			**TEPM**	
			
			**2 h**	**6 h**	**24 h**	**1 h**	**7 h**
**Induced by LPS in BMM and TEPM**							

**Class A**							
	NM_009630	Adora2a	1.2	28.2	3.3	4.2	52.2
	NM_007413	Adora2b	27.4	190.9	17.4	3.0	3.3
	NM_007719	Ccr7	1.0	5.1	1.9	0.4	2.9
	NM_017466	Ccrl2	39.2	65.7	46.7	13.8	9.2
	NM_010336	Edg2	7.3	5.7	0.3	1.0	10.0
	NM_007904	Ednrb	6.7	2.0	10.3	0.7	5.3
	NM_013521	Fpr1	5.9	120.9	23.3	1.0	21.4
	NM_008042	Fprl1	3.8	41.6	28.6	3.1	570.6
	NM_030701	Gpr109b	13.6	48.2	5.6	10.8	212.0
	NM_030720	Gpr84	8.7	10.3	0.2	7.8	47.5
	NM_145066	Gpr85	3.6	5.6	0.5	18.1	4.9
**Class B**							
	NM_018782	Calcrl	1.0	2.6	0.2	0.6	6.9
**Frizzled**							
	NM_021457	Fzd1	8.0	17.0	1.0	1.0	2.2

**Induced by LPS in BMM**							

**Class A**							
	NM_009912	Ccr1	0.9	3.1	7.4	0.8	0.7
	NM_009911	Cxcr4	0.3	0.1	7.2	0.7	0.1
	NM_004230	Edg5	0.2	0.5	2.0	0.4	0.6
	NM_182806	Gpr18	17.8	52.2	10.8	1.0	1.6
	NM_175493	Gpr68	3.3	1.7	40.2	1.5	0.8
	NM_013533	Gpr162	1.1	0.5	6.7	0.8	0.8
	NM_008311	Htr2b	0.6	0.5	15.8	0.6	0.3
	NM_008519	Ltb4r1	0.8	0.8	7.5	0.9	0.7
	NM_008773	P2ry2	3.7	7.5	3.7	1.9	1.1
	NM_021381	Prokr1	0.8	37.4	7.2	0.3	0.1
	NM_008964	Ptger2	2.4	1.3	11.7	0.3	0.1
	NM_008967	Ptgir	1.1	2.3	7.4	0.7	0.6
**Class B**							
	NM_139138	Emr4	1.1	2.0	1.0	1.0	0.9
	NM_001081298	Lphn2	2.2	1.4	1.2	0.3	0.3

**Induced by LPS in TEPM**							

**Class A**							
	NM_007577	C5r1	1.1	0.7	0.1	3.0	1.1
	NM_007722	Cxcr7	0.5	0.5	0.5	1.0	4.0
	NM_133200	P2ry14	0.7	1.7	1.4	0.8	6.7

Micro-array analysis was used to assess the gene expression profile of GPCRs upon LPS treatment in primary mouse macrophages (BMM and TEPM). GPCRs regulated by LPS in primary mouse macrophages were defined as those that were 2-fold or greater up-regulated at 2, 6 or 24 h LPS in BMM and/or 1 or 7 h LPS in TEPM (and significantly different from controls, p-value < 0.05, Student's two-tailed t-test). Values shown depict gene expression relative to untreated control (0 h). GPCRs are grouped according to their class type on the basis of sequence similarity as previously described [65-67].

**Table 4:** GPCRs repressed by LPS in macrophages.

			**Relative Normalised Expression**				
			
**Receptor Class**	**Accession Number**	**Gene Name**	**BMM**			**TEPM**	
			
			**2 h**	**6 h**	**24 h**	**1 h**	**7 h**
**Repressed by LPS in BMM and TEPM**							

**Class A**							
	NM_009924	Cnr2	0.2	0.1	0.3	0.3	0.1
	NM_009911	Cxcr4	0.3	0.1	7.1	0.7	0.1
	NM_183031	Ebi2	0.2	0.1	0.1	0.4	0.2
	NM_004230	Edg5	0.2	0.5	2.0	0.4	0.6
	NM_022320	Gpr35	0.2	0.4	1.6	0.6	0.1
	NM_030258	Gpr146	0.1	0.4	1.0	0.4	0.1
	NM_008772	P2ry1	0.3	0.1	0.2	0.3	0.1
	NM_175116	P2ry5	0.1	0.1	0.7	0.3	0.1
	NM_013641	Ptger1	0.7	0.5	0.3	0.7	0.3
	NM_008965	Ptger4	3.1	0.5	1.0	1.9	0.3

**Repressed by LPS in BMM**							

**Class A**							
	NM_007420	Adrb2	0.3	0.1	0.1	1.9	0.8
	NM_009779	C3ar1	0.7	0.5	0.3	0.8	0.6
	NM_007577	C5r1	1.1	0.7	0.1	3.0	1.1
	NM_009915	Ccr2	0.5	0.4	0.2	0.8	0.9
	NM_009910	Cxcr3	0.7	0.1	0.1	1.6	0.9
	NM_009987	Cx3cr1	0.5	0.1	0.1	1.0	0.7
	NM_030720	Gpr84	8.3	10.0	0.2	7.7	50.0
	NM_027571	P2ry12	0.5	0.1	0.2	1.0	1.0
**Class B**							
	NM_018782	Calcrl	1.0	2.6	0.2	0.6	7.1

**Repressed by LPS in TEPM**							

**Class A**							
	NM_007719	Ccr7	1.0	5.0	1.9	0.4	2.9
	NM_021381	Prokr1	0.8	33.3	7.1	0.3	0.1
	NM_198168	Ppp2r5b	1.0	1.3	1.1	0.8	0.5
	NM_008964	Ptger2	2.4	1.3	11.1	0.3	0.1
**Class B**							
	NM_080437	Celsr3	0.8	1.2	2.3	0.2	0.2
	NM_173036	Gpr97	0.8	1.0	0.8	0.5	0.2
	NM_016894	Ramp1	1.0	1.0	1.0	0.5	0.2
**Class C**							
	NM_022420	Gprc5b	1.0	1.0	1.3	0.8	0.1

Micro-array analysis was used to assess the gene expression profiles of GPCRs upon LPS treatment in primary mouse macrophages (BMM and TEPM). GPCRs regulated by LPS in primary mouse macrophages were defined as those that were 2-fold or greater down-regulated at 2, 6 or 24 h LPS in BMM and/or 1 or 7 h LPS in TEPM (and significantly different from controls, p-value < 0.05, Student's two-tailed t-test). Values shown depict gene expression relative to untreated control (0 h). GPCRs are grouped according to their class type on the basis of sequence similarity as previously described [65-67].

The LPS time courses studied in BMM (2, 6 and 24 h) and TEPM (1 and 7 h) were not identical and so it is difficult to make definitive conclusions about non-conserved regulation by LPS in these two cell populations. Nonetheless, we did identify genes that appeared to be differentially regulated by LPS in BMM or TEPM at similar time points. *Gpr18*, *P2ry2*, *P2ry12 *and *Adrb2 *were all strongly regulated by LPS in BMM, but not TEPM (Table 3, 4), whilst LPS regulated *Gprc5b *expression in TEPM, but not BMM (Table 4). The effects of LPS could even be opposing in these two cell populations; *Prokr1 *was induced 37-fold by LPS in BMM (6 h), but repressed 20-fold in TEPM (7 h) (Figure 3c). Again some of these differences may be explained by the proliferative state of the cells; LPS triggers growth arrest in BMM and so some of the LPS-regulated genes unique to BMM may reflect this phenomenon. This is clearly not the case for all genes however (e.g. *P2ry14 *and *Prokr1*), thus arguing that subtle differences in LPS signaling pathways may exist in BMM versus TEPM.

## Discussion

With an understanding of the GPCRs that are (a) highly expressed by the mononuclear phagocyte system, (b) selectively expressed by certain macrophage populations, or (c) regulated by the pro-inflammatory stimulus LPS, one can make some inferences about the role of specific GPCRs in macrophage biology. One of the most obvious findings from our analysis was that many of the P2Y purinergic receptors, which detect purine and pyrimidine nucleotides, were expressed in a restricted and/or regulated fashion in macrophages. Mechanical stress, cellular injury, inflammatory stimuli such as LPS, degranulation of mast cells and hypoxia can all increase extracellular concentrations of ATP and UTP [17-20]. Consequently, these receptors are very likely to impact upon macrophage-mediated inflammatory responses.

Of the P2Y family, perhaps most striking in its expression pattern was *P2ry6*, which was essentially restricted to cells of the myeloid lineage. *P2ry6 *mRNA levels were greatly elevated in BMM, TEPM, RAW264 cells, microglia, osteoclasts and dendritic cells, compared to all other cell types (Figure 1b). *P2ry6 *expression in osteoclasts, which are a closely related lineage to macrophages, has been confirmed by others [21]. Whilst no previous studies have indicated that *P2ry6 *expression is restricted to the monocyte/macrophage lineage, there is evidence to suggest that this receptor does regulate the function of macrophages. UDP, which is reportedly a selective P2RY6 agonist [22,23], triggered interleukin (IL)-8 release from the human monocyte-like cell line, THP-1 [24]. Conversely, the P2RY6 receptor antagonists, anthraquinone-sulfonic acid derivative reactive blue 2 and suramin, inhibited LPS-induced IL-8 production by THP-1 cells [24]. In microglia, *P2ry6 *mRNA expression was up-regulated in response to neuronal damage and UDP promoted phagocytosis by microglia, thus implying a role for P2RY6 in the clearance of damaged or dead neuronal cells [23]. Our observation that this receptor is widely expressed in the mononuclear phagocyte system suggests that this receptor may have a broader function in sensing and engulfing damaged cells throughout the body, as well as promoting pro-inflammatory responses in response to LPS and/or UDP.

This laboratory previously reported that *P2ry5 *mRNA expression was dramatically repressed by LPS in human and mouse macrophages [2], a finding supported by micro-array data presented here. This receptor was originally classified as a member of the P2Y receptor family on the basis of sequence identity and its ability to bind the nucleotides ATP and ADP [25]. Nucleotides failed to initiate downstream signaling from this receptor however [26], and it is therefore possible that P2RY5 acts as an antagonist of other P2Y receptors and restricts the macrophage response to extracellular nucleotides. Down-regulation of P2RY5 in response to a macrophage activating stimulus such as LPS might remove this inhibitory effect, thus allowing activated macrophages to respond to nucleotides. Alternatively, it is possible that the initial assignment of P2RY5 as a P2 receptor was incorrect. Cross-genome phylogenetic analysis showed that it clustered with a heterogenous group of receptors including the protease-activated receptors, as well as lysophosphatidylcholine (LPA) and sphingosylphosphorylcholine (S1P) receptors, rather than with other P2Y receptors [27]. In this case, LPS-mediated macrophage activation would presumably render cells unresponsive to the true ligand for this receptor.

Emerging evidence also implicates other P2Y receptors in macrophage-mediated inflammatory responses. For example, extracellular ATP, an agonist of P2RY2, induced IL-6 transcription in human monocyte-derived macrophages [28] and chemotaxis in human neutrophils [29], while the P2RY1 agonist 2-methylthio ATP increased IL-6 secretion from mouse splenocytes [30]. In support of a role for P2Y ATP receptors in macrophage function, and consistent with our data, *P2ry1 *and *P2ry2 *mRNAs were expressed at high levels in macrophages and LPS-stimulated monocytes [31]. Apart from these P2Y family members, P2RY12 was also reported to be selectively expressed in microglia in the rat brain [32]. Our analysis also showed that this receptor was highly enriched in microglia, as well as BMM, plasmacytoid dendritic cells, osteoclasts and a variety of lymphoid and neural tissues [see additional file 2a]. Finally, the P2Y-like receptor, P2ry14 (Gpr105), which is a receptor for UDP-conjugated sugars, mediated chemotaxis of bone marrow hematopoietic stem cells [33]. Despite the emerging literature that suggests a role for P2Y receptors in macrophage-mediated migration and inflammatory responses, a clear understanding of the selective agonists of these receptors and their interplay with each other and non-P2Y receptors is lacking. Nonetheless, our demonstration of the restricted and regulated expression of this family in macrophages identifies them as promising candidates for further analysis.

Other GPCRs that had a relatively restricted expression pattern and were strongly expressed by macrophages include *Edg5*, *Gpr85 *and *Gpr84 *(Table 2, Figure 1a and 1c, Additional file 2b). *Edg5 *is one of eight members of the endothelial differentiation gene (EDG) family of GPCRs that recognize LPA and S1P [34]. Several studies have documented potent effects of the EDG5 ligand, S1P on macrophages. Rat alveolar macrophages responded to S1P by producing O_2_^- ^at levels comparable to those induced by LPS or formyl-Methionyl-Leucyl-Phenylalanine [35]. Not surprisingly, given this finding, anti-microbial effects of S1P have been reported. In human monocyte-derived macrophages or THP-1 cells infected with *M. smegmatis *or *M. tuberculosis*, S1P reduced intracellular bacterial loads in a dose-dependent manner. Similar effects of S1P were also apparent in mice infected with *M. smegmatis *or *M. tuberculosis *[36]. Apart from activating anti-microbial responses, S1P also provided a survival signal to both mouse and human macrophage cell lines upon subsequent exposure to an apoptosis-inducing signal [37]. EDG1 was implicated in this response, but given the high expression of *Edg5 *reported here, this receptor may also be involved in macrophage responses to S1P.

Expression of the SREB (super conserved receptor expressed in brain) family member *Gpr85 *(*Sreb2*) was restricted to macrophages and neuronal tissues (Figure 1a). Whilst the expression of *Gpr85 *in the central nervous system was previously reported [38-40], a function has yet to be identified. The co-regulated expression of receptors such as *Gpr85 *in neural tissues and leukocyte populations may provide a mechanism for the neuro-immune cross-talk that has been observed in several studies [41,42].

*Gpr84 *is an orphan GPCR that was strikingly restricted to BMM and microglia in an unstimulated state (Table 2, Figure 1c) and was strongly up-regulated by LPS in both BMM and TEPM (Figure 3a, Table 3). This up-regulation is consistent with elevated *Gpr84 *expression in microglia and tissue macrophages in an endotoxin shock model [43]. Microglia also expressed high levels of *Gpr84 *in the experimental autoimmune encephalomyelitis model of multiple sclerosis [43]. These data imply that GPR84 has a role in neuroinflammation, but the exact function is difficult to predict without any knowledge of a ligand. Nonetheless, given the highly restricted expression pattern of *Gpr84 *mRNA, antibodies against this receptor may provide useful tools for tracking macrophage populations *in vivo*.

As with *Gpr84*, mRNA for several other GPCRs, particularly chemokine receptors, were differentially expressed between various unstimulated macrophage populations. TEPM expressed elevated levels of mRNA for *Cxcr4*, *Ccr1*, *Ccrl2*, whilst BMM expressed higher levels of *Ccr2*, *Cx3cr1 *and *Cxcr3*. The fractalkine receptor CX3CR1 has been identified as a marker of patrolling monocytes that survey the endothelial surface [44] and its down-regulation on macrophages that have already trafficked to an inflammatory site is not surprising. Conversely, CXCR4 has a well-characterized function in targeting leukocytes and their progenitors away from the circulation [45], consistent with its enhanced expression in TEPM. Apart from chemokine receptors, the expression of a number of other GPCRs was different between BMM and TEPM. For example, the thrombin-related receptor, *Ebi2 *[46], was strongly expressed in BMM but not TEPM, whilst *Gprc5b *was enriched in TEPM but not BMM. Such differences are likely to contribute to functional divergence between tissue macrophage and inflammatory macrophage populations.

Many of the GPCRs that were highly expressed by macrophages in an unstimulated state (described above), were also regulated by LPS. Several other GPCRs were weakly expressed by unstimulated macrophages but were strongly regulated by LPS. One such example was the frizzled family member, *Fzd1*. Frizzleds (Fzd) represent the cell membrane receptors for a family of secreted glycoproteins called wingless-related proteins (WNTs). Wnts play essential roles in development, including cell fate determination, adhesion, polarity, migration and proliferation [47-50]. The WNT family has also been implicated in immune regulation. For example, the WNT family member, WNTD, acted as a negative feedback regulator of Toll signaling in *Drosophila *[51]. In human macrophages, WNT5A expression was up-regulated by TLR ligands, as well as challenge with *M. tuberculosis *and *M. avium*, and the WNT5A/Frizzled-5 (FZD5) pathway promoted IL-12 production from peripheral blood mononuclear cells [52]. The striking up-regulation of *Fzd1 *in BMM (17-fold at 6 h LPS) implies that this FZD family member shares similar functions in regulating macrophage inflammatory responses.

We also identified several GPCRs that were differentially regulated by LPS in BMM and TEPM. In BMM, *Prokr1 *was transiently induced by LPS (37-fold at 6 h) but repressed in TEPM (20-fold at 7 h). PROKR1 is a receptor for prokineticin 1 (PK1), a peptide that regulates monocyte differentiation, as well as macrophage activation and migration. PK1 triggered the differentiation of both murine and human bone marrow progenitor cells into the monocyte/macrophage lineage [53], and reprogrammed the response of human monocytes to LPS by amplifying IL-12 and tumor necrosis factor alpha (TNF) production, but inhibiting IL-10 release [53]. The differential regulation of *Prokr1 *by LPS in BMM versus TEPM implies that this receptor has distinct functions during activation of tissue versus inflammatory macrophage populations.

*Gpr18 *was induced 52-fold in BMM following 6 h LPS exposure, while it remained unchanged in TEPM. This receptor was also highly expressed in other immune cell populations including B cells, T cells and DC cells [see additional file 2c]. N-arachidonylglycine (NAGly), a conjugate of arachidonic acid and glycine, is an endogenous ligand for GPR18 [54] that was reported to suppress inflammatory pain and have analgesic properties [55,56]. Given the potent regulation of *Gpr18 *expression in BMM by LPS, NAGly is also likely to regulate macrophage function. The adenosine receptor, *Adora2b *was also strongly induced by LPS in BMM (191-fold at 6 h) yet was only modestly regulated (3-fold at 7 h) in TEPM. Adenosine-mediated activation of this receptor augmented IL-10 production by RAW264 cells in response to LPS [57]. In contrast, adenosine attenuated LPS-induced production of the pro-inflammatory cytokines TNF [58-61] and IL-12 [60,62] in mouse and human macrophages. Inducible *Adora2b *expression may therefore contribute to feedback regulation of macrophage inflammatory responses.

## Conclusion

In this study, we have documented several GPCRs that are detectably expressed in primary mouse macrophages (BMM, TEPM), enriched in either or both BMM and TEPM, or regulated in activated macrophages (LPS-stimulated BMM and/or TEPM). For some of these genes, constitutive or regulated expression in macrophages has not previously been described. Future studies on such GPCRs and their agonists are likely to provide important insights into macrophage biology, as well as novel inflammatory pathways which could be future targets for drug discovery.

## Methods

### Cell culture and reagents

All cell lines and tissues were sourced from 8–10 week old male C57Bl/6 mice, with the exception of female-specific organs, which were sourced from female mice. All procedures were carried out in accordance with local guidelines for animal research. For female tissues, material was pooled from three females and for each female an average four embryos resulting in four umbilical cords and placentas were obtained. For other tissues, material was derived from a pool of three males. Biological replicates were defined as independent RNA preparations from independent pools of mice. Technical replicates were defined as independent amplifications from the same RNA sample. The full list of cell lines and tissues profiled, as well as further details on biological versus technical replication, is provided in additional file 3 (key to figures 1, 2 and additional file 2).

Mouse BMM were prepared from bone marrow cells from femurs of adult C57Bl/6 mice by culturing for 7 days in complete medium (RPMI containing 10% FCS, 20 U/ml penicillin, 20 μg/ml streptomycin and 2 mM L-glutamine (Invitrogen) in the presence of 10 000 U/ml CSF-1 (a gift from Chiron, Emeryville, CA) on bacteriological plastic plates. On day 7, BMM were treated with 10 ng/mL *Salmonella minnesota *LPS (Re595 mutant, Sigma) for 2, 6 or 24 h, and RNA was extracted. To generate TEPM, cells were harvested from the peritoneal cavity of C57Bl/6 mice 3 d after i.p. administration of 3% thioglycollate broth (Difco). Cells were then plated onto tissue culture plastic for 2 h, after which time non-adherent cells were removed by washing with PBS. Adherent TEPM were then treated with LPS for 1 or 7 h, or were left untreated, and RNA was extracted. Microglia were generated from cultures of mixed cortical cells. After 10 d culture, microglia were separated from astroglia by shaking on an orbital shaker (180 rpm, 20 min, 37°C) in the presence of 12 mM lidocaine (Sigma).

### RNA and cDNA preparation

RNA extraction from mammalian cells or tissues was performed using RNeasy kits (Qiagen) or the standard Trizol protocol. If tissue amounts were more than 50 mg per mouse when dissected, tissues were pulverized while frozen. RNA was prepared separately for every mouse in order to identify samples with potentially degraded RNA. Trizol-extracted RNA was purified with a Qiagen RNeasy column. For BMM, osteoblasts and osteoclasts contaminating genomic DNA was removed during the RNeasy cleanup using DNaseI (Qiagen). The integrity and concentration of RNA was determined via microfluidic analysis on a bio-analyser (Agilent Technologies) or by analysis on a BioRad Experion (BioRad). Pooling occurred at the RNA level. For samples containing more than two μg total RNA available after pooling, standard Affymetrix single amplification was performed using two μg total RNA. For pooled samples containing less than two μg total RNA, 100 ng total RNA (or 50 ng for thymocyte SP CD8+) was used in a standard Affymetrix double amplification protocol. The starting quantity of RNA for specific tissues is included in additional file 3.

### Microarray analysis

Standard Affymetrix protocols were used to process Affymetrix MOE430_2 microarrays. All CEL file images were processed as a single group using gcRMA [63,64], after which the data for each gene was rescaled using per-gene normalisation to the median (for tissue specificity analysis) or per-gene normalisation to untreated control samples (for analysis of BMM and TEPM gene regulation by LPS). The microarray data described have been deposited in NCBIs Gene Expression Omnibus [5] and are accessible through GEO Series accession number GSE10246. GPCRs identified as highly enriched in BMM or TEPM were defined as transcripts where the normalised expression value was as least 10-fold greater in BMM/TEPM when compared to the median expression across all cell types and where BMM/TEPM expression significantly deviated from this median value (p-value < 0.05, student's two-tailed t-test). GPCRs regulated by LPS in primary mouse macrophages were defined as those whose normalised expression values were two-fold or greater up- or down-regulated at any point in the time course (2, 6 or 24 h LPS in BMM and/or 1 or 7 h LPS in TEPM) compared to the unstimulated state (0 LPS). LPS regulated genes were also filtered on the basis of statistical significance (p-value < 0.05, student's two-tailed t-test). All statistical testing was performed using the GeneSpring GX software, with the cross-gene error model function enabled.

## Abbreviations

BMM: bone marrow-derived macrophage; EDG: endothelial differentiation gene; Fzd: Frizzled; GPCR: G Protein-Coupled Receptor; IL: interleukin; LPA: lysophosphatidylcholine; LPS: lipopolysaccharide; NAGly: N-arachidonylglycine; P2Y: purinergic receptor family Y; PK1: prokineticin 1; S1P: sphingosylphosphorylcholine; SREB: super conserved receptor expressed in brain; TEPM: thioglycollate-elicited peritoneal macrophage; TLR: Toll-like Receptor; TNF: tumor necrosis factor alpha; Wnt: Wingless-related protein.

## Competing interests

The authors declare that they have no competing interests.

## Authors' contributions

JL carried out filtering and analysis of the GNF microarray expression data and drafted the manuscript. KS carried out the extraction of the microarray expression data and participated in the design of the study and review of the manuscript. AS, JZ and JW generated GNF microarray expression data. KS and CG purified cell populations and generated RNA for microarray analysis. DH and SK assisted in the review and editing of the manuscript. MS participated in the design of the study and drafting of the manuscript. All authors read and approved the final manuscript.

## Supplementary Material

Additional file 1 GPCR expression in Mouse Macrophages. The table lists all GPCRs expressed in mouse macrophages. Click here for file

Additional file 2 Macrophage-restricted GPCRs. The data provided demonstrates the macrophage-restricted expression of *P2ry12*, *Edg5 *and *Gpr18 *mRNA. Micro-array analysis of *P2ry12 *(A), *Edg5 *(B) and *Gpr18 *(C) mRNA expression across a panel of 91 murine cell types and tissues. Data points show normalised values and similar cell types are grouped according to bar colour; blue indicates primary macrophage cell types, purple indicates bone-related cell types, red indicates other immune cell types, green indicates stem cell populations, orange indicates whole tissue samples, yellow indicates neuronal and retinal cell types and pink indicates cell lines. Additional file 3 gives details of the 91 cell types and tissues profiled. Click here for file

Additional file 3 Cell panel sample types (legend key for figure 1 and 2 and additional file 1). The table provides a full list of the cell lines and tissues profiled in the micro-array analysis. Click here for file

## References

[B1] McKnight AJ, Macfarlane AJ, Dri P, Turley L, Willis AC, Gordon S (1996). Molecular cloning of F4/80, a murine macrophage-restricted cell surface glycoprotein with homology to the G-protein-linked transmembrane 7 hormone receptor family. J Biol Chem.

[B2] Lattin J, Zidar DA, Schroder K, Kellie S, Hume DA, Sweet MJ (2007). G-protein-coupled receptor expression, function, and signaling in macrophages. J Leukoc Biol.

[B3] Su AI, Wiltshire T, Batalov S, Lapp H, Ching KA, Block D, Zhang J, Soden R, Hayakawa M, Kreiman G, Cooke MP, Walker JR, Hogenesch JB (2004). A gene atlas of the mouse and human protein-encoding transcriptomes. Proc Natl Acad Sci U S A.

[B4] Genomics Institute of the Novartis Research Foundation SA Genomics Institute of the Novartis Research Foundation, [http://symatlas.gnf.org/SymAtlas].

[B5] NCBIs Gene Expression Omnibus NCBIs Gene Expression Omnibus, [http://www.ncbi.nlm.nih.gov/geo/].

[B6] Foord SM, Bonner TI, Neubig RR, Rosser EM, Pin JP, Davenport AP, Spedding M, Harmar AJ (2005). International Union of Pharmacology. XLVI. G protein-coupled receptor list. Pharmacol Rev.

[B7] International Union of Basic and Clinical Pharmacology GPCRRFF International Union of Basic and Clinical Pharmacology, [http://www.iuphar-db.org/GPCR/ReceptorFamiliesForward].

[B8] Austyn JM, Gordon S (1981). F4/80, a monoclonal antibody directed specifically against the mouse macrophage. Eur J Immunol.

[B9] Hirsch S, Austyn JM, Gordon S (1981). Expression of the macrophage-specific antigen F4/80 during differentiation of mouse bone marrow cells in culture. J Exp Med.

[B10] Chenoweth DE, Goodman MG, Weigle WO (1982). Demonstration of a specific receptor for human C5a anaphylatoxin on murine macrophages. J Exp Med.

[B11] Gasque P, Singhrao SK, Neal JW, Wang P, Sayah S, Fontaine M, Morgan BP (1998). The receptor for complement anaphylatoxin C3a is expressed by myeloid cells and nonmyeloid cells in inflamed human central nervous system: analysis in multiple sclerosis and bacterial meningitis. J Immunol.

[B12] Shimada T, Matsumoto M, Tatsumi Y, Kanamaru A, Akira S (1998). A novel lipopolysaccharide inducible C-C chemokine receptor related gene in murine macrophages. FEBS Lett.

[B13] Katsuyama M, Ikegami R, Karahashi H, Amano F, Sugimoto Y, Ichikawa A (1998). Characterization of the LPS-stimulated expression of EP2 and EP4 prostaglandin E receptors in mouse macrophage-like cell line, J774.1. Biochem Biophys Res Commun.

[B14] Mandal P, Novotny M, Hamilton TA (2005). Lipopolysaccharide induces formyl peptide receptor 1 gene expression in macrophages and neutrophils via transcriptional and posttranscriptional mechanisms. J Immunol.

[B15] Schaub A, Futterer A, Pfeffer K (2001). PUMA-G, an IFN-gamma-inducible gene in macrophages is a novel member of the seven transmembrane spanning receptor superfamily. Eur J Immunol.

[B16] Wahl JR, Goetsch NJ, Young HJ, Van Maanen RJ, Johnson JD, Pea AS, Brittingham A (2005). Murine macrophages produce endothelin-1 after microbial stimulation. Exp Biol Med (Maywood).

[B17] Bergfeld GR, Forrester T (1992). Release of ATP from human erythrocytes in response to a brief period of hypoxia and hypercapnia. Cardiovasc Res.

[B18] Bodin P, Burnstock G (1995). Synergistic effect of acute hypoxia on flow-induced release of ATP from cultured endothelial cells. Experientia.

[B19] Bodin P, Burnstock G (1998). Increased release of ATP from endothelial cells during acute inflammation. Inflamm Res.

[B20] Detwiler TC, Feinman RD (1973). Kinetics of the thrombin-induced release of adenosine triphosphate by platelets. Comparison with release of calcium. Biochemistry.

[B21] Korcok J, Raimundo LN, Du X, Sims SM, Dixon SJ (2005). P2Y6 nucleotide receptors activate NF-kappaB and increase survival of osteoclasts. J Biol Chem.

[B22] Communi D, Parmentier M, Boeynaems JM (1996). Cloning, functional expression and tissue distribution of the human P2Y6 receptor. Biochem Biophys Res Commun.

[B23] Koizumi S, Shigemoto-Mogami Y, Nasu-Tada K, Shinozaki Y, Ohsawa K, Tsuda M, Joshi BV, Jacobson KA, Kohsaka S, Inoue K (2007). UDP acting at P2Y6 receptors is a mediator of microglial phagocytosis. Nature.

[B24] Warny M, Aboudola S, Robson SC, Sevigny J, Communi D, Soltoff SP, Kelly CP (2001). P2Y(6) nucleotide receptor mediates monocyte interleukin-8 production in response to UDP or lipopolysaccharide. J Biol Chem.

[B25] Webb TE, Kaplan MG, Barnard EA (1996). Identification of 6H1 as a P2Y purinoceptor: P2Y5. Biochem Biophys Res Commun.

[B26] Li Q, Schachter JB, Harden TK, Nicholas RA (1997). The 6H1 orphan receptor, claimed to be the p2y5 receptor, does not mediate nucleotide-promoted second messenger responses. Biochem Biophys Res Commun.

[B27] Metpally RP, Sowdhamini R (2005). Cross genome phylogenetic analysis of human and Drosophila G protein-coupled receptors: application to functional annotation of orphan receptors. BMC Genomics.

[B28] Hanley PJ, Musset B, Renigunta V, Limberg SH, Dalpke AH, Sus R, Heeg KM, Preisig-Muller R, Daut J (2004). Extracellular ATP induces oscillations of intracellular Ca2+ and membrane potential and promotes transcription of IL-6 in macrophages. Proc Natl Acad Sci U S A.

[B29] Chen Y, Corriden R, Inoue Y, Yip L, Hashiguchi N, Zinkernagel A, Nizet V, Insel PA, Junger WG (2006). ATP release guides neutrophil chemotaxis via P2Y2 and A3 receptors. Science.

[B30] Straub RH, Pongratz G, Gunzler C, Michna A, Baier S, Kees F, Falk W, Scholmerich J (2002). Immunoregulation of IL-6 secretion by endogenous and exogenous adenosine and by exogenous purinergic agonists in splenic tissue slices. J Neuroimmunol.

[B31] Guerra AN, Fisette PL, Pfeiffer ZA, Quinchia-Rios BH, Prabhu U, Aga M, Denlinger LC, Guadarrama AG, Abozeid S, Sommer JA, Proctor RA, Bertics PJ (2003). Purinergic receptor regulation of LPS-induced signaling and pathophysiology. J Endotoxin Res.

[B32] Sasaki Y, Hoshi M, Akazawa C, Nakamura Y, Tsuzuki H, Inoue K, Kohsaka S (2003). Selective expression of Gi/o-coupled ATP receptor P2Y12 in microglia in rat brain. Glia.

[B33] Lee BC, Cheng T, Adams GB, Attar EC, Miura N, Lee SB, Saito Y, Olszak I, Dombkowski D, Olson DP, Hancock J, Choi PS, Haber DA, Luster AD, Scadden DT (2003). P2Y-like receptor, GPR105 (P2Y14), identifies and mediates chemotaxis of bone-marrow hematopoietic stem cells. Genes Dev.

[B34] Takuwa Y, Takuwa N, Sugimoto N (2002). The Edg family G protein-coupled receptors for lysophospholipids: their signaling properties and biological activities. J Biochem (Tokyo).

[B35] Hornuss C, Hammermann R, Fuhrmann M, Juergens UR, Racke K (2001). Human and rat alveolar macrophages express multiple EDG receptors. Eur J Pharmacol.

[B36] Garg SK, Volpe E, Palmieri G, Mattei M, Galati D, Martino A, Piccioni MS, Valente E, Bonanno E, De Vito P, Baldini PM, Spagnoli LG, Colizzi V, Fraziano M (2004). Sphingosine 1-phosphate induces antimicrobial activity both in vitro and in vivo. J Infect Dis.

[B37] Weigert A, Johann AM, von Knethen A, Schmidt H, Geisslinger G, Brune B (2006). Apoptotic cells promote macrophage survival by releasing the antiapoptotic mediator sphingosine-1-phosphate. Blood.

[B38] Hellebrand S, Schaller HC, Wittenberger T (2000). The brain-specific G-protein coupled receptor GPR85 with identical protein sequence in man and mouse maps to human chromosome 7q31. Biochim Biophys Acta.

[B39] Hellebrand S, Wittenberger T, Schaller HC, Hermans-Borgmeyer I (2001). Gpr85, a novel member of the G-protein coupled receptor family, prominently expressed in the developing mouse cerebral cortex. Brain Res Gene Expr Patterns.

[B40] Matsumoto M, Beltaifa S, Weickert CS, Herman MM, Hyde TM, Saunders RC, Lipska BK, Weinberger DR, Kleinman JE (2005). A conserved mRNA expression profile of SREB2 (GPR85) in adult human, monkey, and rat forebrain. Brain Res Mol Brain Res.

[B41] Salzet M (2000). Invertebrate molecular neuroimmune processes. Brain Res Brain Res Rev.

[B42] Ransohoff RM, Liu L, Cardona AE (2007). Chemokines and chemokine receptors: multipurpose players in neuroinflammation. Int Rev Neurobiol.

[B43] Bouchard C, Page J, Bedard A, Tremblay P, Vallieres L (2007). G protein-coupled receptor 84, a microglia-associated protein expressed in neuroinflammatory conditions. Glia.

[B44] Auffray C, Fogg D, Garfa M, Elain G, Join-Lambert O, Kayal S, Sarnacki S, Cumano A, Lauvau G, Geissmann F (2007). Monitoring of blood vessels and tissues by a population of monocytes with patrolling behavior. Science.

[B45] Kucia M, Jankowski K, Reca R, Wysoczynski M, Bandura L, Allendorf DJ, Zhang J, Ratajczak J, Ratajczak MZ (2004). CXCR4-SDF-1 signalling, locomotion, chemotaxis and adhesion. J Mol Histol.

[B46] Birkenbach M, Josefsen K, Yalamanchili R, Lenoir G, Kieff E (1993). Epstein-Barr virus-induced genes: first lymphocyte-specific G protein-coupled peptide receptors. J Virol.

[B47] Clevers H (2006). Wnt/beta-catenin signaling in development and disease. Cell.

[B48] Cohen SM, Di Nardo S (1993). Wingless: from embryo to adult. Trends Genet.

[B49] Dierick H, Bejsovec A (1999). Cellular mechanisms of wingless/Wnt signal transduction. Curr Top Dev Biol.

[B50] Klingensmith J, Nusse R (1994). Signaling by wingless in Drosophila. Dev Biol.

[B51] Gordon MD, Dionne MS, Schneider DS, Nusse R (2005). WntD is a feedback inhibitor of Dorsal/NF-kappaB in Drosophila development and immunity. Nature.

[B52] Blumenthal A, Ehlers S, Lauber J, Buer J, Lange C, Goldmann T, Heine H, Brandt E, Reiling N (2006). The Wingless homolog WNT5A and its receptor Frizzled-5 regulate inflammatory responses of human mononuclear cells induced by microbial stimulation. Blood.

[B53] Dorsch M, Qiu Y, Soler D, Frank N, Duong T, Goodearl A, O'Neil S, Lora J, Fraser CC (2005). PK1/EG-VEGF induces monocyte differentiation and activation. J Leukoc Biol.

[B54] Kohno M, Hasegawa H, Inoue A, Muraoka M, Miyazaki T, Oka K, Yasukawa M (2006). Identification of N-arachidonylglycine as the endogenous ligand for orphan G-protein-coupled receptor GPR18. Biochem Biophys Res Commun.

[B55] Burstein SH, Rossetti RG, Yagen B, Zurier RB (2000). Oxidative metabolism of anandamide. Prostaglandins Other Lipid Mediat.

[B56] Huang SM, Bisogno T, Petros TJ, Chang SY, Zavitsanos PA, Zipkin RE, Sivakumar R, Coop A, Maeda DY, De Petrocellis L, Burstein S, Di Marzo V, Walker JM (2001). Identification of a new class of molecules, the arachidonyl amino acids, and characterization of one member that inhibits pain. J Biol Chem.

[B57] Nemeth ZH, Lutz CS, Csoka B, Deitch EA, Leibovich SJ, Gause WC, Tone M, Pacher P, Vizi ES, Hasko G (2005). Adenosine augments IL-10 production by macrophages through an A2B receptor-mediated posttranscriptional mechanism. J Immunol.

[B58] Bouma MG, Stad RK, van den Wildenberg FA, Buurman WA (1994). Differential regulatory effects of adenosine on cytokine release by activated human monocytes. J Immunol.

[B59] Hasko G, Szabo C, Nemeth ZH, Kvetan V, Pastores SM, Vizi ES (1996). Adenosine receptor agonists differentially regulate IL-10, TNF-alpha, and nitric oxide production in RAW 264.7 macrophages and in endotoxemic mice. J Immunol.

[B60] Link AA, Kino T, Worth JA, McGuire JL, Crane ML, Chrousos GP, Wilder RL, Elenkov IJ (2000). Ligand-activation of the adenosine A2a receptors inhibits IL-12 production by human monocytes. J Immunol.

[B61] McWhinney CD, Dudley MW, Bowlin TL, Peet NP, Schook L, Bradshaw M, De M, Borcherding DR, Edwards CK (1996). Activation of adenosine A3 receptors on macrophages inhibits tumor necrosis factor-alpha. Eur J Pharmacol.

[B62] Hasko G, Kuhel DG, Chen JF, Schwarzschild MA, Deitch EA, Mabley JG, Marton A, Szabo C (2000). Adenosine inhibits IL-12 and TNF-[alpha] production via adenosine A2a receptor-dependent and independent mechanisms. Faseb J.

[B63] Wu Z, Irizarry RA (2005). Stochastic models inspired by hybridization theory for short oligonucleotide arrays. J Comput Biol.

[B64] Zhijin Wu RAI, Francisco Martinez-Murillo FS (2004). A Model Based Background Adjustment for
Oligonucleotide Expression Arrays. Johns Hopkins University, Dept of Biostatistics Working Papers.

[B65] Flower DR (1999). Modelling G-protein-coupled receptors for drug design. Biochim Biophys Acta.

[B66] Gether U, Kobilka BK (1998). G protein-coupled receptors. II. Mechanism of agonist activation. J Biol Chem.

[B67] Pierce KL, Premont RT, Lefkowitz RJ (2002). Seven-transmembrane receptors. Nat Rev Mol Cell Biol.

