# Supplementary material for: Expression analysis of G Protein-Coupled Receptors in mouse macrophages
Source: Immunome Res. 2008 Apr 29;4:5. doi: 10.1186/1745-7580-4-5 (PMC2394514; doi:10.1186/1745-7580-4-5)
Supplement: Additional file 1 — GPCR expression in Mouse Macrophages. The table lists all GPCRs expressed in mouse macrophages. [file 1745-7580-4-5-S1.doc]

## Additional Table 1. GPCR expression in Mouse Macrophages.

| **Receptor Class** | **Accession Number** | **Gene Name** |
| --- | --- | --- |
| **Class A** |  |  |
|  | NM_009630 | Adora2a |
|  | NM_007413 | Adora2b |
|  | NM_007419 | Adrb1 |
|  | NM_007420 | Adrb2 |
|  | NM_016847 | Avpr1a |
|  | NM_030258 | Gpr146 |
|  | NM_009779 | C3ar1 |
|  | NM_007577 | C5r1 |
|  | NM_009912 | Ccr1 |
|  | NM_009915 | Ccr2 |
|  | NM_009917 | Ccr5 |
|  | NM_007719 | Ccr7 |
|  | NM_145700 | Ccrl1 |
|  | NM_017466 | Ccrl2 |
|  | NM_007698 | Chrm1 |
|  | NM_008153 | Cmklr1 |
|  | NM_009924 | Cnr2 |
|  | NM_009987 | Cx3cr1 |
|  | NM_009910 | Cxcr3 |
|  | NM_009911 | Cxcr4 |
|  | NM_007722 | Cxcr7 |
|  | NM_021476 | Cysltr1 |
|  | NM_183031 | Ebi2 |
|  | NM_007901 | Edg1 |
|  | NM_010336 | Edg2 |
|  | [NM_004230](http://www.ncbi.nlm.nih.gov/entrez/viewer.fcgi?val=NM_004230.3) | Edg5 |
|  | NM_007904 | Ednrb |
|  | NM_010170 | F2rl2 |
|  | NM_146187 | Ffar2 |
|  | NM_013521 | Fpr1 |
|  | NM_008042 | Fprl1 |
|  | NM_133200 | P2ry14 |
|  | NM_182806 | Gpr18 |
|  | NM_008157 | Gpr19 |
|  | NM_022320 | Gpr35 |
|  | NM_008152 | Gpr65 |
|  | NM_175493 | Gpr68 |
|  | NM_030720 | Gpr84 |
|  | NM_145066 | Gpr85 |
|  | NM_028808 | Gpr86 |
|  | NM_030701 | Gpr109b |
|  | XM_977547 | Gpr160 |
|  | NM_013533 | Gpr162 |
|  | NM_008311 | Htr2b |
|  | NM_008519 | Ltb4r1 |
|  | NM_008772 | P2ry1 |
|  | NM_008773 | P2ry2 |
|  | NM_175116 | P2ry5 |
|  | NM_183168 | P2ry6 |
|  | NM_027571 | P2ry12 |
|  | [NM_198168](http://www.ncbi.nlm.nih.gov/entrez/viewer.fcgi?val=NM_198168.3) | Ppp2r5b |
|  | NM_021381 | Prokr1 |
|  | NM_013641 | Ptger1 |
|  | NM_008964 | Ptger2 |
|  | NM_008965 | Ptger4 |
|  | NM_008967 | Ptgir |
| **Class B** |  |  |
|  | NM_018782 | Calcrl |
|  | NM_011925 | Cd97 |
|  | NM_080437 | Celsr3 |
|  | NM_010130 | Emr1 |
|  | NM_139138 | Emr4 |
|  | NM_173036 | Gpr97 |
|  | [NM_001081298](http://www.ncbi.nlm.nih.gov/entrez/viewer.fcgi?val=NM_001081298.1) | Lphn2 |
|  | NM_016894 | Ramp1 |
| **Class C** |  |  |
|  | NM_022420 | Gprc5b |
| **Class Frizzled** |  |  |
|  | NM_021457 | Fzd1 |
|  | NM_008057 | Fzd7 |

Micro-array analysis was used to identify GPCRs that were detectable in primary mouse macrophages (BMM and TEPM) either constitutively or upon LPS stimulation. GPCR are grouped according to their class type on the basis of sequence similarity as previously described [64-66].
