# Supplementary material for: Expression analysis of G Protein-Coupled Receptors in mouse macrophages
Source: Immunome Res. 2008 Apr 29;4:5. doi: 10.1186/1745-7580-4-5 (PMC2394514; doi:10.1186/1745-7580-4-5)
Supplement: Additional file 3 — Cell panel sample types (legend key for figure 1 and 2 and additional file 1). The table provides a full list of the cell lines and tissues profiled in the micro-array analysis. [file 1745-7580-4-5-S3.doc]

## Additional Table 2. Cell panel sample types (legend key for figure 1, 2 and additional file 1).

| **Sample Number** | **Sample Type** | **Gender** | **Replicate Type** | **RNA Amount (ng)** | **Amplification Type** |
| --- | --- | --- | --- | --- | --- |
| 1 | BMM unstimulated | male | experimental | 2000 | single |
| 2 | BMM 2hr LPS | male | experimental | 2000 | single |
| 3 | BMM 6hr LPS | male | experimental | 2000 | single |
| 4 | BMM 24hr LPS | male | experimental | 2000 | single |
| 5 | RAW 264.7 | unknown | technical | 2000 | single |
| 6 | TEPM unstimulated | male | technical | 2000 | single |
| 7 | TEPM 1hr LPS | male | technical | 2000 | single |
| 8 | TEPM 7hr LPS | male | technical | 2000 | single |
| 9 | Bone | male | experimental | 2000 | single |
| 10 | Bone marrow | male | experimental | 2000 | single |
| 11 | Microglia | male | experimental | 1000 | single |
| 12 | Osteoclasts | male | experimental | 2000 | single |
| 13 | Osteoblast day5 | male | experimental | 2000 | single |
| 14 | Osteoblast day14 | male | experimental | 2000 | single |
| 15 | Osteoblast day21 | male | experimental | 2000 | single |
| 16 | Mast cells | male | experimental | 100 | double |
| 17 | Mast cells IgE | male | experimental | 100 | double |
| 18 | Mast cells IgE+antigen 1hr | male | experimental | 100 | double |
| 19 | Mast cells IgE+antigen 6hr | male | experimental | 100 | double |
| 20 | B-cells marginal zone | male | technical | 100 | double |
| 21 | Follicular B-cells | male | experimental | 2000 | single |
| 22 | NK cells | male | experimental | 100 | double |
| 23 | T-cells CD4+ | male | technical | 100 | double |
| 24 | T-cells CD8+ | male | technical | 100 | double |
| 25 | T-cells foxP3+ | male | experimental | 100 | double |
| 26 | Dendritic cells lymphoid CD8a+ | male | technical | 100 | double |
| 27 | Dendritic cells myeloid CD8a- | male | technical | 100 | double |
| 28 | Dendritic plasmacytoid B220+ | male | technical | 100 | double |
| 29 | Common myeloid progenitor | male | experimental | 100 | double |
| 30 | Granulo-mono progenitor | male | experimental | 100 | double |
| 31 | Granulocytes_mac1+gr1+ | male | experimental | 100 | double |
| 32 | Mega erythrocyte progenitor | male | experimental | 100 | double |
| 33 | Thymocyte DP CD4+CD8+ | male | technical | 100 | double |
| 34 | Thymocyte SP CD4+ | male | technical | 100 | double |
| 35 | Thymocyte SP CD8+ | male | technical | 50 | double |
| 36 | Stem cells | male | experimental | 100 | double |
| 37 | Embryonic stem line Bruce4 p13 | unknown | technical | 2000 | single |
| 38 | Embryonic stem line V26 2 p16 | unknown | technical | 2000 | single |
| 39 | Adipose brown | male | experimental | 2000 | single |
| 40 | Adipose white | male | experimental | 2000 | single |
| 41 | Bladder | male | experimental | 2000 | single |
| 42 | Epidermis | male | experimental | 2000 | single |
| 43 | Heart | male | experimental | 2000 | single |
| 44 | Intestine large | male | experimental | 2000 | single |
| 45 | Intestine small | male | experimental | 2000 | single |
| 46 | Kidney | male | experimental | 2000 | single |
| 47 | Lacrimal gland | male | experimental | 2000 | single |
| 48 | Liver | male | experimental | 2000 | single |
| 49 | Lung | male | experimental | 2000 | single |
| 50 | Lymph nodes | male | experimental | 2000 | single |
| 51 | Mammary gland lact | female | experimental | 2000 | single |
| 52 | Mammary gland non-lactating | female | technical | 2000 | single |
| 53 | Ovary | female | experimental | 2000 | single |
| 54 | Pancreas | male | experimental | 2000 | single |
| 55 | Pituitary | male | experimental | 2000 | single |
| 56 | Placenta | female | experimental | 2000 | single |
| 57 | Prostate | male | experimental | 2000 | single |
| 58 | Salivary gland | male | experimental | 2000 | single |
| 59 | Skeletal muscle | male | experimental | 2000 | single |
| 60 | Spleen | male | experimental | 2000 | single |
| 61 | Stomach | male | experimental | 2000 | single |
| 62 | Testis | male | experimental | 2000 | single |
| 63 | Umbilical cord | female | experimental | 2000 | single |
| 64 | Uterus | female | experimental | 2000 | single |
| 65 | Iris | male | experimental | 100 | double |
| 66 | Cornea | male | technical | 100 | double |
| 67 | Eyecup | male | technical | 100 | double |
| 68 | Lens | male | technical | 100 | double |
| 69 | Retina | male | technical | 100 | double |
| 70 | Retinal pigment epithelium | male | experimental | 100 | double |
| 71 | Olfactory bulb | male | experimental | 2000 | single |
| 72 | Adrenal gland | male | experimental | 2000 | single |
| 73 | amygdala | male | experimental | 2000 | single |
| 74 | cerebellum | male | experimental | 2000 | single |
| 75 | Cerebral cortex | male | experimental | 2000 | single |
| 76 | Cerebral cortex prefrontal | male | experimental | 2000 | single |
| 77 | Dorsal root ganglia | male | experimental | 2000 | single |
| 78 | Dorsal striatum | male | experimental | 2000 | single |
| 79 | Ciliary bodies | male | technical | 100 | double |
| 80 | Hippocampus | male | experimental | 2000 | single |
| 81 | Hypothalamus | male | experimental | 2000 | single |
| 82 | Neuro2a | unknown | technical | 2000 | single |
| 83 | Nucleus_accumbens | male | experimental | 2000 | single |
| 84 | Spinal cord | male | experimental | 2000 | single |
| 85 | 3T3-L1 | unknown | technical | 2000 | single |
| 86 | Baf3 | unknown | technical | 2000 | single |
| 87 | C2C12 | unknown | technical | 2000 | single |
| 88 | C3H 10T1 2 | unknown | technical | 2000 | single |
| 89 | mIMCD-3 | unknown | technical | 2000 | single |
| 90 | NIH3T3 | unknown | technical | 2000 | single |
| 91 | Min6 | unknown | technical | 2000 | single |

Full list of the cell lines and tissues profiled in the micro-array analysis. The gender, replicate type, starting concentration of RNA and the amplification type are also indicated. Biological replicates were defined as RNA from two separate pools from independent mice. Technical replicates were defined as the same total RNA from a pool of three mice which was split for two different amplifications. Standard Affymetrix single amplification was performed for samples for which there was at least two µg total RNA available after pooling, while a standard Affymetrix double amplification protocol using 100 ng total RNA (or 50 ng for thymocyte_SP_CD8+) was used for samples where less than two µg total RNA was available.
